# Psoriasis-Like Inflammation Induced Renal Dysfunction through the TLR/NF-*κ*B Signal Pathway

**DOI:** 10.1155/2020/3535264

**Published:** 2020-01-21

**Authors:** Fang Ren, Min Zhang, Caiyun Zhang, Hong Sang

**Affiliations:** ^1^Department of Dermatology, Jinling Hospital, School of Medicine, Nanjing University, 305# Zhongshan East Road, Nanjing, Jiangsu 210003, China; ^2^Department of Dermatology, The Affiliated Jiangning Hospital of Nanjing Medical University, 168# Gushan Road, Nanjing, Jiangsu 211100, China

## Abstract

Pathological studies have shown an association between psoriasis and renal injury (RI), but the mechanism between RI and psoriasis was still unclear. This paper was designed to investigate the relationship and mechanism between psoriasis-like inflammation and renal injury in BALB/C mice. Mice were topically smeared imiquimod followed by various analyses in skin lesions, urine protein, kidney/serum inflammatory cytokines, kidney function, podocyte membrane proteins, and toll-like receptors/nuclear factor kappa-b (TLR/NF-*κ*B) pathway-associated proteins. Meanwhile, lipopolysaccharide (LPS) and dexamethasone (DEX) were intraperitoneally injected to promote and inhibit inflammation accompanied by imiquimod to elaborate the relevance between inflammatory levels and RI. In the model group, the Psoriasis Area and Severity Index (PASI) scores of scaly and erythema obviously increased (*p* < 0.01), creatinine and blood urea nitrogen significantly increased (*p* < 0.01), the positive area of hematoxylin-eosin (HE) and periodic acid-Schiff (PAS) staining in kidney increased (*p* < 0.01), malondialdehyde significantly increased with superoxide dismutase (SOD) decreased (*p* < 0.01), 24-hour urine protein increased and the expressions of podocin and CD2 associate protein (CD2AP) decreased (*p* < 0.01), and kidney/serum inflammatory factors (IL-17, IL-1*β*, IL-6, TNF-*α*, and IL-22) and TLR/NF-*κ*B-related expression (TLR2, TLR4, MyD88, and NF-*κ*Bp65) all increased (*p* < 0.01). The RI was aggravated with the TLR/NF-*κ*B related expression being upregulated by LPS (*p* < 0.05). On the contrary, the RI was alleviated by DEX (*p* < 0.05). Our data showed that psoriasis-like inflammation damaged the renal function via the TLR/NF-*κ*B signal pathway. Inhibiting TLR/NF-*κ*B-related protein expression may be effective for the treatment of RI caused by psoriasis.

## 1. Introduction

Psoriasis was a chronic, immune-mediated inflammatory dermatosis, which affected nearly 2-3% of the general population [1]. Psoriasis caused systemic inflammation, increased oxidative stress, and eventually produced many complications, such as hypertension, diabetes, liver disease, and atherosclerosis [2, 3]. Moderate-to-severe psoriasis associated with chronic kidney disease was independent of traditional risk factors [4, 5]. Although there was a correlation between renal dysfunction and psoriasis in some clinical research, the potential mechanism associated with psoriasis and renal dysfunction was rarely explored.

Many inflammatory cytokines, such as tumor necrosis factor-*α* (TNF-*α*), interleukin- (IL-) 17, IL-1, were elevated in the skin of psoriasis. Meanwhile, the serum concentration of these inflammatory cytokines was also associated with the severity of psoriasis [6]. It was proved that IL-23/T help (Th)-17 immune axis, IL-1 and TNF-*α* played a central role in the pathogenesis of psoriasis. These cytokines interacted to promote the production and development of psoriasis-like inflammation [7–9]. TNF antagonists, IL-17, and IL-12/23 inhibition with monoclonal antibodies were the main treatment measures of psoriasis [10]. Therefore, what this research focuses on was the relationship between psoriasis-like inflammation and renal injury.

It was reported that the increased levels of inflammatory factors induced podocyte injury and the production of proteinuria, deteriorating to renal dysfunction [11–14]. The transcription and release of proinflammatory cytokines such as IL-1*β*, IL-6, and TNF-*α* in podocytes promoted inflammatory response and led to podocyte injury [15–18]. Such as in the diabetic hyperglycemic environment, the body's inflammation levels significantly increased, which promoted podocyte injury and ultimately manifested as diabetic nephropathy [19, 20].

Therefore, we hypothesized that high expressions of renal inflammatory cytokines causes podocyte injury and eventually manifest as renal dysfunction. To verify this hypothesis, we established psoriasis-like model in BALB/C mice. Skin lesions, inflammatory factors, antioxidant markers, proteinuria, renal function, microstructural changes of kidneys, the expression of CD2AP and podocin proteins, and the expression of TLR/NF-*κ*B pathway-associated proteins were detected subsequently. Furthermore, lipopolysaccharide (LPS) and dexamethasone (DEX) groups were, respectively, carried out to upregulate and downregulate inflammatory levels during the experiment. Finally, the mechanism of inflammatory-mediated psoriasis nephropathy and its related signaling pathways was revealed.

## 2. Materials and Methods

### 2.1. Animals

Male SPF grade BALB/C mice (10–12 weeks of age; 25–30 g) were provided by the Experimental Animal Center of Jiangsu University (Zhenjiang, China) and the certificate of conformity is SYXK (Su) 20130036. The mice were housed in separate cages, alternating for 12 h light/dark cycle and keeping the surrounding temperature at 24–26°C with a humidity of 40%. The mice drank water freely and fed standard ordinary feed. All animal experiments were approved by the Animal Ethics Committee.

### 2.2. Reagents and Instruments

Ultrapure water was prepared by a Milli-Q50 SP Reagent Water System (Millipore Corporation, MA, USA). The M-MLV reverse transcription kit was obtained from Invitrogen Corporation (Thermo Fisher Science, MA, USA). Ethanol, chloroform, and isopropanol (China National Pharmaceutical Group, Beijing, CHN); rabbit anti-mice podocin polyclonal antibody (Abcam, Cambs, UK); triazole reagent (Thermo Fisher Science, MA, USA); rabbit anti-mice CD2AP polyclonal antibody (Bioss Antibodies, Beijing, CHN); primers (Genewiz, NJ, USA); LPS (Bellancom Beijing, CHN); and DEX (Guangxi Wonder Pharmacy Stock Co. Ltd, Guangxi, CHN). The cDNA first-strand synthesis kit and SYBR green master mix were purchased from Novozymes (Novozymes, Beijing, CHN); hydrated chloral (Huabo Instrument Co. Ltd, Shanxi, CHN); creatinine kit (Jiancheng Bioengineering Institute, Nanjing, CHN); TLR, p-IkB*α*, IkB*α*, p-NF-kBp65, p-IKK*β*, KK*β*, and NFkBp65 (Cell Signaling Technology, MA, USA) were purchased.

The analytical balance version is BSA224S-CW (Sartorius, Niedersachsen, Germany). The pipette version is Finnpipette F3 (Thermo Fisher Science, MA, USA). The enzyme label version is 9602 (Perlong Medical, Beijing, CHN). The fluorescent quantitative PCR circulator version is 7500 (the ABI, MA, USA). The desktop-refrigerated microspeed centrifuge version is Micro7R (Thermo Fisher Science, MA, USA). The fully automatic sample fast grinding machine version is JXFSTPRP-64 (Shanghai Jingxin Industrial Development Co. Ltd, Shanghai, CHN). The UV spectrophotometer version is Evolution 300 PC (Thermo Fisher Science, MA, USA). The gel imaging system version is BioDoc-It220 (the UVP, CA, USA). The common PCR instrument version is TCA5020 (Thermo Fisher Science, MA, USA). Nucleic acid electrophoresis apparatus version is DYCP-35 (Liuyi, Beijing, CHN).

### 2.3. Group and Administration

After 2 days of adaptation period, the BALB/C mice were shaved on the back with a depilatory cream. Then, 5% imiquimod cream (50 mg) was applied once a day for successive 7 days to create psoriasis-like model [21, 22]. Mice were divided into the following groups with 10 in each group: blank group, model group, LPS group, and DEX group. Among them, the blank group was smeared with petrolatum and injected intraperitoneally normal saline (NS) for 7 days. In the model group, NS was injected intraperitoneally after using 5% imiquimod cream for 7 days. The LPS group was smeared with 5% imiquimod and injected intraperitoneally LPS (0.1 mg/kg) for 7 days. The DEX group was smeared with 5% imiquimod and followed by intraperitoneal injection of DEX (0.1 mg/kg) for 7 days.

### 2.4. Evaluating Indicators

#### 2.4.1. Skin Lesions

After 7 consecutive days of administration, the erythema and scaly in skin lesions of four groups were evaluated and calculated by the PASI score.

#### 2.4.2. Urine Protein in 24 Hours

The mice were placed in the metabolic cage, and their urine was collected to determine their content of 24-hour urine protein according to the instructions and procedures of ELISA kits.

#### 2.4.3. Renal Function

To investigate the changes of renal function in all groups of mice, creatinine (Cre) and blood urea nitrogen (BUN) in mice serum were mainly investigated by ELISA kit.

#### 2.4.4. Inflammatory Cytokines in the Serum and Kidneys

After 7 days of continuous administration, mice serum samples were obtained by eyeball sampling, followed by centrifugation at 5000 rpm for 10 min, and the supernatant was taken. Then, the mice were sacrificed by cervical dislocation. The bilateral kidneys were removed, homogenized, and centrifuged at 10,000 rpm for 10 minutes. The inflammatory response-related cytokines (IL-1*β*, IL-6, TNF-*α*, IL-22, and IL-17) in the serum and kidneys were analyzed by ELISA kits. The specific operation procedures refer to the instructions attached to the kit.

#### 2.4.5. Antioxidant Indexes in Kidneys

After 7 days of continuous administration, the mice were sacrificed, and the bilateral kidneys were removed and homogenized. After centrifugation at 10,000 rpm for 10 min, the appropriate amount of supernatant was taken, and the contents of SOD and malondialdehyde (MDA) were detected by ELISA.

#### 2.4.6. Renal Pathological Examination

The renal cortex was fixed with formaldehyde (10%) for 24 hours and washed with water and dehydrated with 70%, 80%, 90%, 95%, and 100% ethanol gradient for 30 min. We used xylene to transparent, then dipped wax, embedded in paraffin, and cut into slices. The slices were about 2 *μ*m in thickness and stained with hematoxylin-eosin (HE) and periodic acid-Schiff stain (PAS) separately. Pathological changes of the kidneys were observed under light microscope.

#### 2.4.7. CD2AP and Podocin Protein Expression in Kidneys

Western blotting: 60 mg of renal tissue was taken and RNA was extracted with protein lysate RIPA. Protein concentration was detected with Bradford protein quantitation kit. 30 *μ*g samples of each group were taken separately, adding the total protein sample, and the buffer solution of the protein gel electrophoresis. Then, they were mixed well, denatured at 95°C for 10 min, and putted in ice-bath. The samples (30 *μ*g) were slowly added into the gel hole and passed through the concentration gel and separation gel (voltage 8 V/cm) under a stable voltage condition of 80 V. The dye was electrophoresed to a suitable position in the separation gel, and the sample was transferred into the polyvinylidene fluoride (PVDF) membrane on ice. The PVDF membrane with protein transfer was blocked with 5% skim milk powder at 4°C, and rabbit anti-mice podocin primary antibody (1 : 1000) and rabbit anti-mice CD2AP primary antibody (1 : 300) were added. Then, wash the membrane with TBST and put the washed primary antibody reaction membrane into the secondary antibody working solution (1 : 5000). Slowly shake for 60 min under the condition of avoiding light and room temperature. Then, membranes were washed, reacted with TMB method, and exposed. The gray ratio of target protein/*β*-actin indicates the relative amount of target protein.

#### 2.4.8. Expressions of TLR/NF-*κ*B Pathway-Related Proteins

During the extraction of renal protein, lysate was added. After ice bath for 30 min, it was centrifuged at 12,000 r/m for 6 min (completed at 4°C). The total protein concentration was measured using the quinolinic protein assay kit. The polyacrylamide gel electrophoresis process was described as previously, and the protein expression levels were quantified using image analysis software (Image J 5.0 software).

### 2.5. Statistical Analysis

Experimental data were expressed as mean ± standard deviation, and one-way analysis of variance (ANOVA) was performed using SPSS 19.0 statistical analysis software. Statistical significance was indicated by *p* < 0.05.

## 3. Results

### 3.1. Skin Lesions

**Figure 1:**
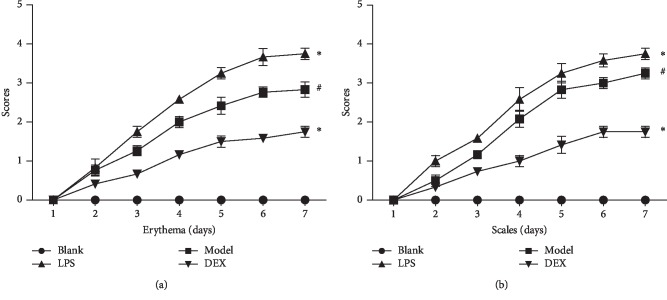
The PASI scores of skin lesions (erythema and scaly) in psoriasis-like mice ($\bar{x}$ ± SD, *n* = 6**).** PASI scores of (a) erythema and (b) scaly. ^#^*p* < 0.01 compared with blank; ^*∗*^*p* < 0.05 compared with model.

In the blank group, their back skins were smooth without desquamation, thick, and erythema. In the model group (psoriasis-like mice), on the 2^nd^ day after the application of imiquimod, their back skins appeared light red. On the 3^rd^ day, their skins were thickened with some erythema and scales. From the 4^th^ to 5^th^ day, the mice had more erythema and scaling with the skin gradually thickening. From the 6^th^ to 7^th^ day, the typical psoriasis-like lesions appeared under the intervention of imiquimod. To the 7^th^ day, the PASI scores of erythema in blank, model, LPS, and DEX were 0.00, 2.75, 4.00, and 2.00, respectively, the PASI scores of erythema in blank, model, LPS, and DEX were 0.00, 3.50, 4.00, and 2.00, respectively (Figure 1).

### 3.2. 24-Hour Urine Protein Content

**Figure 2:**
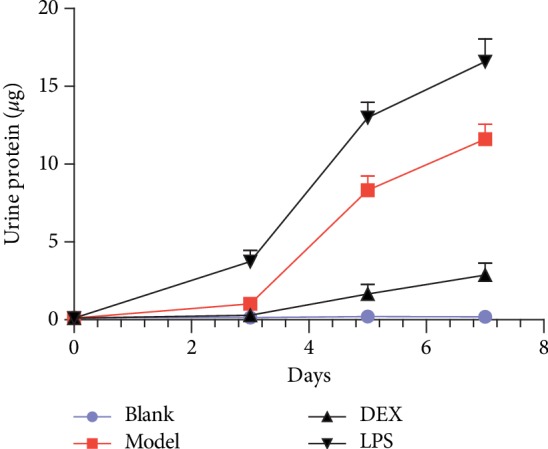
Quantification of 24 h urine protein at different time points of psoriasis-like mice ($\bar{x}$ ± SD, *n* = 6).

To the 7th day, compared with the blank group (0.20 ± 0.16 *μ*g), the content of 24-hour urinary protein was significantly higher in the model group (11.62 ± 0.94 *μ*g, *p* < 0.01). Compared with the model group, the content was significantly increased accompanied after the injection of LPS (16.58 ± 1.46 *μ*g, *p* < 0.05). Meanwhile, the 24-hour urine protein content was significantly decreased under the effect of DEX (2.88 ± 0.76 *μ*g, *p* < 0.05) after 4 weeks. The detailed result was in Figure 2.

### 3.3. The Effect of Psoriasis-Like Response on Renal Function

**Figure 3:**
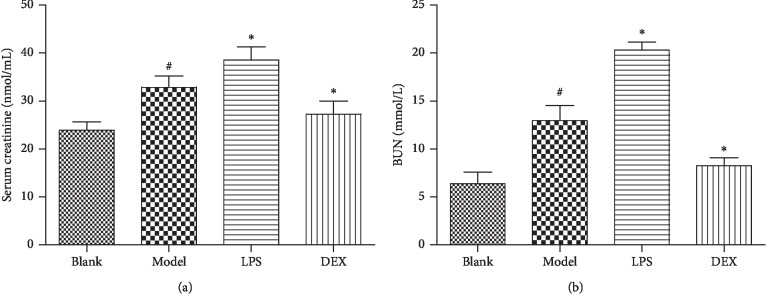
Serum Cre and BUN levels in psoriasis-like mice ($\bar{x}$ ± SD, *n* = 6). ^#^*p* < 0.01 compared with blank; ^*∗*^*p* < 0.05 compared with model.

Through the measurement of Cre and BUN, biological indexes related to renal function, the contents of Cre and BUN in the model group were significantly increased after imiquimod treatment for 7 days compared with the blank group (*p* < 0.01). Compared with the model group, the contents of Cre and BUN were further increased in the LPS group (*p* < 0.05), indicating that the renal injury aggravated after using LPS to enhance inflammatory stimulation. The contents of Cre and BUN were significantly decreased in the DEX group (*p* < 0.05), indicating that renal injury induced by the psoriasis-like reaction was certainly inhibited after using DEX to suppress inflammatory stimulation. The specific result was shown in Figure 3.

### 3.4. Inflammatory Cytokines in the Serum and Kidneys

**Figure 4:**
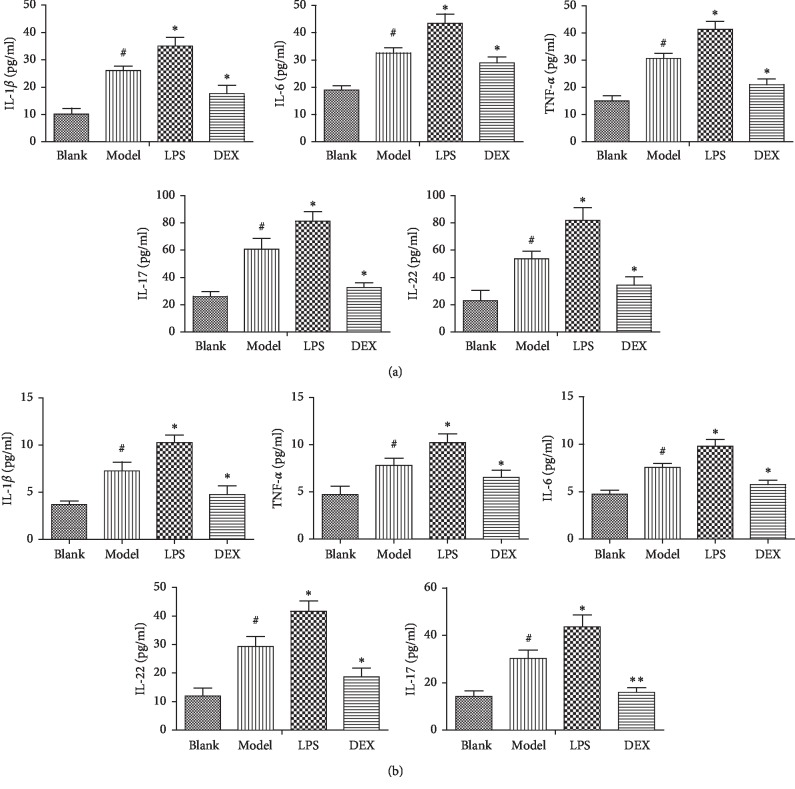
The concentrations of inflammatory factors in the serum (a) and renal (b) of psoriasis-like mice ($\bar{x}$ ± SD, *n* = 6). ^#^*p* < 0.01 compared with blank; ^*∗*^*p* < 0.05 compared with model; ^*∗∗*^*p* < 0.01 compared with model.

Compared with the blank group, the expression of IL-1*β*, IL-6, TNF-*α*, IL-17, and IL-22 were significantly increased in the serum and kidneys of the model group (*p* < 0.01), indicating that the psoriasis-like model was successfully established after using imiquimod and induced psoriasis-like inflammatory response. Compared with the model group, the expressions of IL-1*β*, IL-6, TNF-*α*, IL-17, and IL-22 were continuously increased accompanied by the intraperitoneal injection of LPS (*p* < 0.05). In the DEX group, the expressions of IL-1*β*, IL-6, TNF-*α*, IL-17, and IL-22 were significantly lower than the model group (*p* < 0.05), suggesting that the expressions of inflammatory cytokines associated with psoriasis have been inhibited after the intervention of DEX (Figures 4(a) and 4(b)).

### 3.5. The Antioxidant Capacity

**Figure 5:**
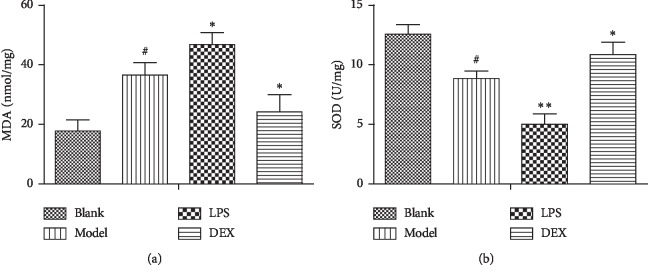
MDA content and SOD activity in the renal of psoriasis-like mice ($\bar{x}$ ± SD, *n* = 6). ^#^*p* < 0.01 compared with blank; ^*∗*^*p* < 0.05 compared with model; ^*∗∗*^*p* < 0.01 compared with model.

Compared with the blank group, the MDA level in the kidneys was significantly increased, and the SOD activity was significantly decreased (*p* < 0.01) in the model group. In the LPS group, the MDA content was significantly increased (*p* < 0.01), and the SOD activity was significantly decreased (*p* < 0.05) compared with the model group. In the DEX group, the MDA content was significantly lower than the model group (*p* < 0.05), and the SOD activity was significantly higher than the model group (*p* < 0.05). The specific result was shown in Figure 5.

### 3.6. Renal Pathological Examination

#### 3.6.1. PAS Staining

**Figure 6:**
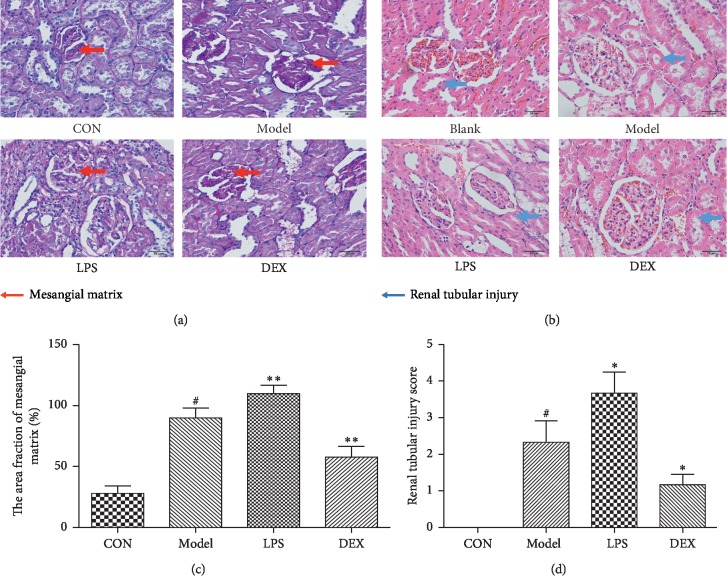
Renal pathological microstructure in psoriasis-like mice ($\bar{x}$ ± SD, *n* = 6). (a) PAS staining. (b) HE staining. ^#^*p* < 0.01 compared with blank; ^*∗*^*p* < 0.05 compared with model; ^*∗∗*^*p* < 0.01 compared with model.

Compared with the blank group, the fraction of mesangial matrix area (%) in the model group was significantly increased (*p* < 0.01). Compared with the model group, the LPS further increased the fraction of mesangial matrix area (*p* < 0.01), inducing the enlargement of the glomerular volume, the thickening of the basement membrane and the mesangial hyperplasia. However, the DEX obviously decreased the fraction of mesangial matrix area (*p* < 0.01), inhibiting and improving glomerular volume enlargement, basement membrane thickening and mesangial hyperplasia (Figures 6(a) and 6(b)).

#### 3.6.2. HE Staining

The normal and clear renal structure, regular globules, regular renal tubules arrangement, and normal morphology of the tubular epithelial cells were observed in the blank group under the light microscopy without atrophy, hypertrophy, thickening of the basement membrane, hyperplasia of the mesangium, expansion of capillary lumen. Compared with the blank group, the renal tubule injury score of the model group significantly increased (*p* < 0.01). Compared with the model group, the renal tubule injury score was significantly reduced in the DEX group (*p* < 0.05) and further aggravated in the LPS group (*p* < 0.01, Figures 6(c) and 6(d)).

### 3.7. Expressions of CD2AP and Podocin in Kidneys

**Figure 7:**
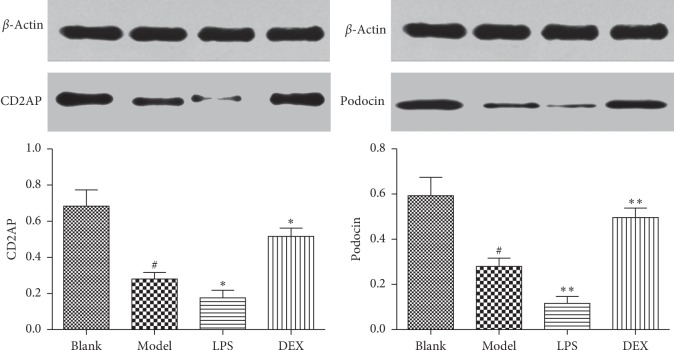
Expression of podocin and CD2AP protein in psoriasis-like mice ($\bar{x}$ ± SD, *n* = 6). ^#^*p* < 0.01 compared with blank; ^*∗*^*p* < 0.05 compared with model; ^*∗∗*^*p* < 0.01 compared with model.

As shown in Figure 7, compared with the blank group, the expression of podocin (0.28 ± 0.05) and CD2AP (0.28 ± 0.04) protein decreased in the renal tissue of the model group (*p* < 0.01). In the LPS group, the expression of podocin (0.12 ± 0.03) and CD2AP (0.18 ± 0.04) protein were lower than the model group (*p* < 0.05). The expression of podocin (0.50 ± 0.04) and CD2AP (0.52 ± 0.05) protein in the DEX group were significantly increased (*p* < 0.05).

### 3.8. Expression of TLR/NF-*κ*B Pathway-Associated Proteins

**Figure 8:**
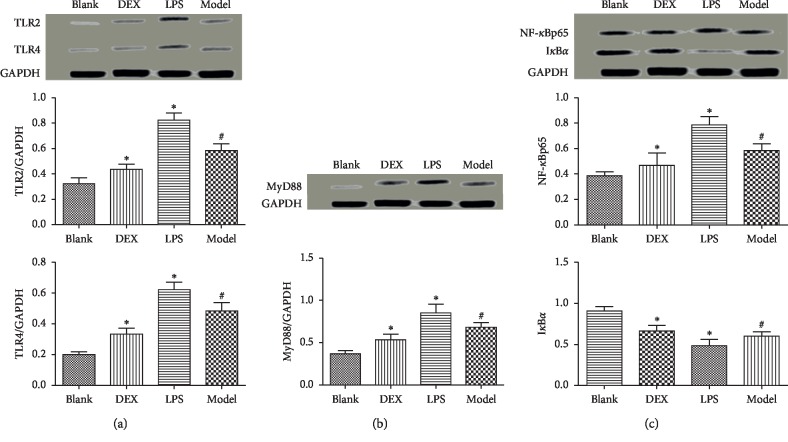
Effect of psoriasis on the expression of TLR/NF-*κ*B pathway-related protein ($\bar{x}$ ± SD, *n* = 6). ^#^*p* < 0.01 compared with blank, ^*∗*^*p* < 0.05 compared with model.

Western blotting results (Figure 8) showed that the expression of TLR/NF-*κ*B pathway-associated proteins in kidneys of the model group were significantly higher (I*κ*B*α* negative correlation) than that in the blank group (*p* < 0.01). Compared with the model group, the LPS continuously promoted the TLR/NF-*κ*B pathway-associated protein expression (*p* < 0.05), and the DEX significantly inhibited TLR/NF-*κ*B pathway-related protein expression (*p* < 0.05).

## 4. Discussion

In the model group, renal function-related indicators (Cre and BUN) were significantly increased that showed the decline of renal function. And, renal pathological lesions such as glomerular volume enlargement, basement membrane thickening, and mesangial hyperplasia were examined by HE and PAS staining techniques. Meanwhile, the expression of inflammatory factors (IL-1*β*, IL-6, TNF-*α*, IL-17, and IL-22) in serum and kidney tissue of psoriasis-like mice was significantly increased. All the results suggested that high expressions of renal inflammatory cytokines caused podocyte injury and eventually manifested as renal dysfunction.

To clarify the relationship between psoriasis nephropathy and inflammation, we added the inflammation promoter (LPS) group and the inflammation inhibitor (DEX) group to investigate the intervention of inflammation on psoriasis nephropathy. Finally, LPS increased the levels of inflammatory factors in serum and kidney, promoted the NF-*κ*Bp65, MyD88, TLR2, and TLR4 protein expression in kidney, inhibited the I*κ*B*α* protein expression, and aggravated the renal injury. DEX reduced the level of inflammation, inhibited the protein expression of NF-*κ*Bp65, MyD88, TLR2, and TLR4 in the kidney, promoted the protein expression of I*κ*B*α* in the kidney of mice, and improved the indicators of kidney function. In previous studies, LPS activated toll-like receptor 4 (TLR4) and then triggered the production of MyD88- and TRIF-dependent proinflammatory cytokines and interferon [23–25]. DEX inhibited TLR4/NF-*κ*Bp65 pathway and the increase of proinflammatory cytokines [26, 27].

TLR was a family of innate immune recognition receptors and played an important role in initiating innate immune responses and inflammatory responses [28]. TLR4 was expressed in the kidney, and its expression was significantly increased in renal tubular epithelial cells and infiltrating leukocytes during renal injury [29]. NF-*κ*B was responsible for the transcription of genes encoding many proinflammatory cytokines and chemokines, and TLR4 was a key regulator of the proinflammatory transcription factor NF-*κ*B [30, 31]. IKK*α* and IKK*β* were the two subtypes of complex IKKs. The upstream effect IKKs activated *κ*B*α* and *κ*B*α* reregulated NF-*κ*B. Once triggered by TLR4, both IKK*α* and IKK*β* phosphorylate, causing I*κ*B*α* to be activated. Afterwards, I*κ*B was phosphorylated and degraded. Excessive TLR caused excessive activation of MyD88 and NF-*κ*B. Additionally, most cytokine gene promoters or enhancers had NF-*κ*B binding sites. Therefore, important cytokines such as TNF-*α*, IL-1*β*, and IL-6 were regulated by NF-*κ*B [32, 33]. The production of these cytokines promoted the occurrence and enhancement of inflammation [34, 35]. TNF-*α* altered renal hemodynamics and nephron transport and affected the transporter activity and expression [36]. IL-1*β* could cause renal injury and nephritis [37, 38]. The abnormal secretion of IL-6 promoted the proliferation of mesangial cells and stroma, thereby affecting the permeability of glomerular filtration membranes [39]. At the same time, T-helper cells 17 (Th 17) could secrete IL-17, IL-22, IL-23, and other cytokines. These cytokines were also involved in the body's inflammatory response inducing the renal injury [40–42]. Therefore, detecting the changes of related cytokines such as IL-6, TNF-*α*, and IL-22 in the serum and kidneys was important to judge the inflammatory cytokines-induced renal injury.

Podocytes were important filtration barriers of the glomerulus, which attached to the highly differentiated epithelial cells outside the glomerular basement membrane. Changes in podocyte structure and function were the main causes of proteinuria [43]. In addition, podocytes played an important role in the occurrence and development of renal injury and were also important indicators for inspection [44]. The nephrin-CD2AP-podocin complex was a key functional unit that constitutes a septal membrane and was an important condition for maintaining the glomerular filtration function [45]. CD2AP was another important protein that maintains the function and structure of podocyte membranes [46]. The denaturation and content changes of these proteins caused defects in signal conduction, leading to podocyte dysfunction, which leaded to progressive glomerulosclerosis and renal failure [47]. Therefore, the expression of podocin and CD2AP protein in renal tissue was an important index to evaluate the degree of renal injury.

**Figure 9:**
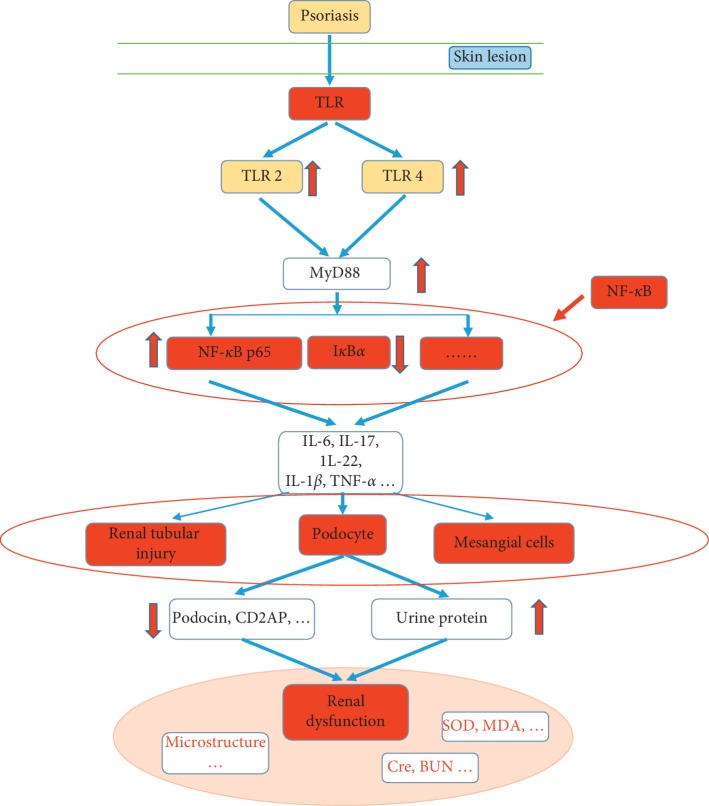
Mechanism of psoriasis-like inflammation-induced renal dysfunction through the TLR/NF-*κ*B signal pathway.

Our data showed that psoriasis-like skin diseases activated TLR receptors based on skin lesions, which increased the expression of TLR2 and TLR4, and then activated MyD88 receptors and promoted the expression of MyD88 protein. MyD88 protein further activated the expression of NF-*κ*B proteins, which increased the expression of NF-*κ*Bp65 protein, decreased the expression of I*κ*B*α* protein, and increased levels of inflammatory factors such as IL-1*β*, IL-6, TNF-*α*, IL-17, and IL-22. The significantly enhanced inflammatory response damaged renal tubules and glomerular cells, main podocytes, and mesangial cells in the kidney and eventually produced renal injury. The detailed pathological mechanism can be seen in Figure 9. Psoriasis-like dermatosis induced an increase in TLR receptor expression, and triggered a subsequent series of inflammatory reactions and kidney damage.

In conclusion, psoriasis induced the expression of inflammatory cytokines, resulting in podocyte injury and aggravating renal injury. On the contrary, inhibiting the expression of inflammatory factors reduced podocyte injury and improved psoriatic renal injury. This study provided important evidence for the clinical prevention and treatment of psoriasis nephropathy.

## Data Availability

The data used to support the findings of this study are available from the corresponding author upon request.
